# Ethnicity and elevated liver transaminases among newly diagnosed children with type 2 diabetes

**DOI:** 10.1186/1471-2431-12-174

**Published:** 2012-11-07

**Authors:** Omar D Hudson, Martha Nunez, Gabriel Q Shaibi

**Affiliations:** 1 Division of Pediatric Endocrinology and Diabetes, Phoenix Children’s Hospital, Phoenix, AZ, USA; 2 Center for Metabolic and Vascular Biology, Arizona State University; 3 College of Nursing and Health Innovation, Arizona State University, 500 N. 3rd St., Phoenix, AZ, 85004, USA

**Keywords:** Type 2 diabetes mellitus, Fatty liver, Alanine aminotransferase, Aspartate aminotransferase

## Abstract

**Background:**

To examine the influence of ethnicity on liver transaminases among adolescents with type 2 diabetes mellitus (T2DM).

**Methods:**

A retrospective medical chart review of 57 (30 males and 27 females) newly diagnosed patients with T2DM. Ethnicity was determined by self-report and height, weight, body mass index (BMI) and glycosylated hemoglobin (HbA1c) were obtained using standard clinical procedures. Fasting levels of alanine aminotransaminase (ALT) and aspartate aminotransferase (AST) were collected prior to the initiation of any therapy.

**Results:**

Age, gender, height, weight, BMI, and HbA1c did not differ between ethnic groups. Compared to African-Americans, Hispanics had significantly higher ALT (23.9 ± 3.4 vs. 107.8 ± 20.3, p=0.002) and AST (17.7 ± 2.5 vs. 71.1 ± 15.7, p<0.001) and were significantly more likely to have ALT values above the upper limit of normal (20% vs. 71%, p=0.005) and twice the upper limit of normal (0% vs. 39%, p=0.05) as well as AST values above the upper limit of normal (0% vs. 53%, p=0.002). No differences in ALT or AST were found between Hispanics and non-Hispanic whites or between African-Americans and non-Hispanic whites.

**Conclusions:**

These preliminary findings suggest that Hispanic children with T2DM may be at higher risk for developing non-alcoholic fatty liver disease and indicate that a comprehensive hepatic evaluation is warranted in this population. Future studies that incorporate more precise and proximal measures of liver health are warranted in this population.

## Background

Childhood obesity is increasing worldwide and nearly 35% of children and adolescents in the United States are overweight or obese [1]. Coinciding with this epidemic is an increase in obesity-related metabolic comorbidities such as type 2 diabetes mellitus (T2DM) and non-alcoholic fatty liver disease (NAFLD) [2,3]. These chronic conditions present challenges to pediatric practitioners as no consensus on their management exists and there is limited evidence regarding long-term prognosis. Both conditions appear to be related to the underlying pathophysiologic process of insulin resistance [4,5] and may act synergistically to place youth at increased risk for premature morbidity and mortality. Only 1 study to date has examined NAFLD risk in youth with T2DM and found that as many as 48% of those diagnosed with T2DM also exhibit elevated liver transaminases [6]. The authors concluded that the high prevalence of elevated serum aminotransferases among children with T2DM may be an indication of concomitant NAFLD in this population.

Although pediatric obesity has increased worldwide, certain ethnic subgroups of youth are disproportionately impacted by both NAFLD and T2DM. In particular, Hispanic and African-American adolescents are more likely to present with T2DM compared to non-Hispanic whites but Hispanic adolescents are thought to be at highest risk for NAFLD while African-Americans are at lowest risk [7]. To date, no studies have examined the degree to which liver transaminases are differentially elevated across ethnic groups among youth diagnosed with T2DM. Given the disproportionate burden of obesity-related disease among minority youth, arriving at a better understanding of NAFLD risk among youth with T2DM is an important step for improving care and management of this vulnerable population. Therefore, the purpose of this preliminary study was to compare serum liver transaminase levels as markers of NAFLD risk in African-American, Hispanic, and non-Hispanic white youth diagnosed with T2DM and assess whether the degree to which these markers are elevated differs by ethnicity.

## Methods

### Patients

The medical charts of patients seen with T2DM in the Division of Pediatric Endocrinology at Phoenix Children’s Hospital between 2005 and 2006 were reviewed. The diagnosis of T2DM was based on the American Diabetes Association criteria in those with negative islet cell, GAD-65 and insulin antibodies [8]. Those diagnosed with T2DM based on known secondary complications of disease or medications (e.g., cystic fibrosis, organ transplant anti-rejection treatment, chemotherapy) or presumed liver disease secondary to viral illnesses, genetic diseases of the hepatobiliary system or autoimmune diseases were excluded. Also excluded were patients in whom the method of diagnosis was not clear or those with insufficient metabolic or ethnicity data available. The Scientific Review Committee and Institutional Review Boards of Phoenix Children’s Hospital approved this study.

### Data collection

The following was extracted from the medical charts: age, gender, ethnicity, height, weight, BMI, HbA1C, and fasting liver transaminases (ALT and AST). All laboratory values were determined at the time of diagnosis prior to the initiation of therapy.

### Data analysis

Data are reported as percentages for categorical variables (gender, ethnicity) or means ± standard error for continuous variables (age, height, weight, body mass index (BMI), ALT, AST, and HbA1c). General linear modeling was used to compare metabolic variables between ethnic groups with Bonferroni adjustments for multiple comparisons. Transaminases were log transformed for normalization however un-transformed values are presented for ease of interpretation. Chi-Squared analyses with Fisher exact test were performed to examine whether the incidence of transaminases above the upper limit of normal (ULN) (ALT ≥ 35 IU/L, AST ≥ 41 IU/L), twice the ULN (ALT ≥ 70 IU/L, AST ≥ 82 IU/L) and three times the ULN (ALT ≥ 105 IU/L, AST ≥ 123 IU/L) differed by ethnicity based on normal values provided by the clinical lab (Quest Diagnostics Inc, Nichols Institute, San Clemente, CA). Statistical significance was determined based on a P value of < 0.05. SPSS statistics 18.0 was used to analyze the data (SPSS Inc, Chicago, IL).

## Results

### Patients

Fifty-seven patients had pertinent metabolic and ethnicity data available and were included in the present analysis. Descriptive characteristics are presented in Table 1. Thirty-eight patients (66%) were identified as Hispanic, 10 (17%) were African-American and 9 (16%) were non-Hispanic white. No significant ethnic differences were noted in age, gender, height, weight, BMI, or HbA1C.

**Table 1:** Descriptive characteristics by ethnicity

	**Hispanic**	**African-American**	**Non-Hispanic White**
Age at Diagnosis (yrs)	13.1 ± 0.3	13.8 ± 0.6	13.5 ± 01.1
Gender (M/F)	23/15	1/9	6/3
Height (cm)	161.7 ± 2.4	160.7 ± 3.8	162.5 ± 5.7
Weight (kg)	90.9 ± 11.0	92.6 ± 9.4	95.7 ± 11.0
BMI (kg/m^2^)	35.0 ± 1.1	38.9 ± 3.3	35.5 ± 3.5
HbA1C (%)	8.9 ± 0.9	9.6 ± 0.4	8.0 ± 0.9

Data expressed as means ± SEM. Abbreviations: BMI (body mass index).

### Liver transaminase data

**Figure 1:**
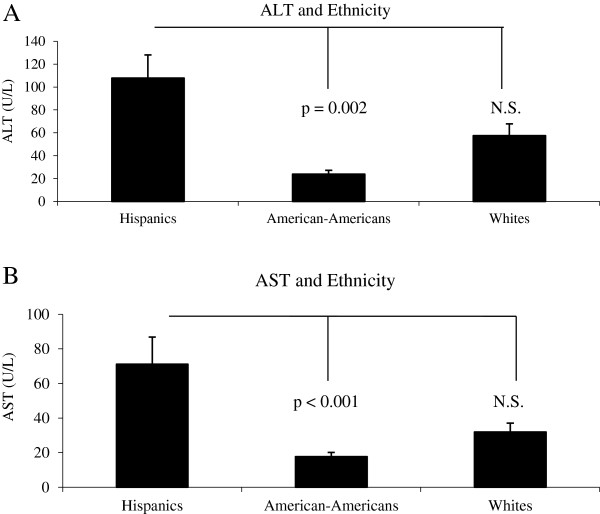
**AB, alanine aminotransferase (ALT) and aspartate aminotransferase (AST) values in newly diagnosed youth with type 2 diabetes mellitus (T2DM).** Data are presented as means ± SE.

ALT and AST data are presented in Figure 1. Both ALT and AST differed by ethnicity (R^2^ = 0.20, F = 6.768, p = 0.002 for ALT and R^2^ = 0.24, F = 8.804, p < 0.001 for AST) with pair-wise comparisons showing Hispanic youth with significantly higher ALT (p = 0.002) and AST (P < 0.001) levels compared to African-Americans. No significant differences were noted between Hispanics and non-Hispanic whites for either ALT (p=1.0) or AST (p=0.36). Similarly, no significant differences were noted between non-Hispanic whites and African-Americans for either ALT (p=0.09) or AST (p=0.18). Ethnicity remained a significant predictor of both ALT and AST after including BMI and HbA1c in the models (F=3.487, p=0.04 and F=3.557, p=0.04, respectively).

The prevalence of elevated transaminases above the ULN, twice the ULN and three times the ULN by ethnicity are presented in Table 2. Hispanics were significantly more likely to have ALT levels above the ULN and twice the ULN (p = 0.008 and 0.05, respectively) with a trend towards significance for having ALT levels 3 times the ULN (p = 0.08) compared to African-Americans or non-Hispanic whites. Additionally, Hispanics were more likely to have AST levels above the ULN (p = 0.003) compared to other groups.

**Table 2:** Percentage of patients with elevated liver transaminases by ethnicity

	**Hispanic (%)**	**African-American (%)**	**Non-Hispanic White (%)**	**P-value**
ALT - Normal	29	80	12	
Above the ULN	71	20	88	0.008
Twice the ULN	39	0	22	0.05
Three times the ULN	29	0	11	0.09
AST - Normal	47	100	89	
Above the ULN	53	0	11	0.003
Twice the ULN	21	0	0	0.17
Three times the ULN	8	0	0	0.45

ALT: Alanine aminotransferase, AST: aspartate aminotransferase, ULN: Upper Limit of Normal.

## Discussion

With the increased prevalence of childhood obesity noted in recent decades, NALFD has become the most common liver abnormality found among the pediatric population [3]. Moreover, NAFLD risk is disproportionately higher among Hispanic youth where elevated ALT levels have been reported in more than 11% of Hispanic adolescents in the US [9], almost 24% of overweight Hispanic youth [10], and as many as 60% of obese Hispanic youth in a clinical sample [11]. The current report extends the available science regarding NAFLD-related health disparities to newly diagnosed adolescents with T2DM and suggest that more than 70% of Hispanic youth with T2DM exhibit elevated ALT levels. These data support recent findings that youth with T2DM are at considerable risk for premature morbidity and mortality [12].

NAFLD and T2DM are thought to share a common pathophysiology related to insulin resistance [13]. Obese youth with NAFLD are significantly more insulin resistant than obese youth who do not exhibit NAFLD [14]. Similarly, adolescents with T2DM are significantly more insulin resistant than BMI-matched adolescents with normal glucose tolerance [4]. Although obesity and insulin resistance are common characteristics of youth with both NAFLD and T2DM, these traits alone do not explain why Hispanic patients in our study exhibited higher transaminase values. Our patients exhibited similar descriptive and glycemic profiles in terms of obesity and severity of diabetes (i.e. similar BMI and HbA1c) which, suggests that ethnicity may further play a role in the pathogenesis of NAFLD in the obese diabetic population. Goran and colleagues [15] have reported that Hispanic and African-American youth are significantly more insulin resistant compared to non-Hispanic whites. Interestingly, the authors noted that the associated compensatory response to insulin resistance is different between these minority groups and may indicate ethnic-specific mechanisms underlying obesity-related diseases. Unfortunately, glucose and insulin levels were not available in our sample, limiting our ability to examine whether variations in insulin resistance may contribute to the observed differences in elevated transaminases between groups. A study by Schwimmer et al [16] identified an extremely high heritability estimate (h^2^ = 0.850; standard error, 0.325), for fatty liver disease in a predominantly Hispanic cohort suggesting that Hispanics may be predisposed to developing NAFLD. This same group hypothesized that African-Americans may either have a protective factor or lack a vulnerability factor for the development of fatty liver [17]. Collectively, these findings suggest that genetic factors may contribute to the higher risk of NAFLD among Hispanic youth. In particular, when coupled with obesity, severe insulin resistance, and T2DM, Hispanic youth may be at extreme risk for premature hepatic morbidity.

The retrospective nature of this report precludes causal conclusions regarding ethnic-specific disease mechanisms or trajectories. A recent study in a community-sample of Korean adults suggests that elevations in ALT predict the development of T2DM over time [18]. A study in obese youth from the United Kingdom suggests that youth with the metabolic syndrome and those with a family history of T2DM, both of which are risk factors for developing T2DM, are more likely to exhibit elevated ALT levels [19]. Therefore, a prospective study following normoglycemic youth as well as patients with T2DM over time or through a course of treatment may better identify potential causal relationships between hepatic health and T2DM or whether gene-environment interactions concomitantly impact the development NAFLD and T2DM. Although our sample had fewer African-Americans and non-Hispanic whites compared to Hispanics this is most likely a function of the ethnic disparities in T2DM risk coupled with the demographics of the local Phoenix metropolitan area. Most of the patients resided in Maricopa County Arizona where Phoenix Children’s Hospital is the only tertiary care referral center for Pediatrics. The most recent census data (2010) showed Hispanics comprise 40.8% of the population while African-Americans comprised 6.5%. These data coupled with the demographics of the patient population in our study support disparities in T2DM risk in the local community and may partially explain the limited number of African-Americans compared to Hispanics and non-Hispanic whites. We acknowledge the limited sample size of the entire cohort and especially the non-Hispanic groups. To our knowledge, the only other publication to date examining liver enzymes in youth with T2DM described 48 youth from Colorado in whom 65% (n=31) had liver enzymes measured at diagnosis [6]. By comparison, the multi-center SEARCH for Diabetes in Youth Study representing 6-centers from four geographically defined populations of 3.5 million youth identified a total of 446 African-American, Hispanic, and non-Hispanic White youth with T2DM in their initial year [20]. Given the paucity of published data in youth with T2DM and the fact that our report is the first to describe the impact of ethnicity on NAFLD in youth with T2DM we expect that future studies will build upon these findings using more representative samples.

It is noteworthy that elevated liver transaminases only appear to be indicators of liver inflammation and are not diagnostic of NAFLD [7]. However, in follow-up of six Hispanic patients with ALT or AST more than twice the ULN who underwent liver ultrasound, characteristics consistent with fatty liver disease were found in all six. Additionally, two of these patients had splenomegaly on the ultrasound with follow up biopsy confirming fibrosis (Grade III in a 15 y/o Hispanic male and Grade II in a 16 year old Hispanic female). The remaining four were followed by both the endocrinology and gastroenterology services. Future studies should employ more sophisticated imaging methods such as magnetic resonance spectroscopy to better quantify liver fat and NAFLD in youth with T2DM [21]. Not only will these studies provide more definitive information on ethnic differences in NAFLD risk, but may also be useful in developing clinical prediction models for when and who to refer for consult with a hepatologist.

As the prevalence of obesity increases in the pediatric population it could be argued that all high-risk patients including Hispanics and especially those newly diagnosed with T2DM have liver transaminases evaluated. In support of this argument, the American Medical Association recently released expert committee recommendations regarding pediatric obesity which suggested biannual screening for NAFLD by transaminase evaluation starting at 10 years of age for children with a BMI ≥95th percentile and those with a BMI between the 85th to 94th percentile who have other risk factors [22]. Furthermore, the committee suggested that ALT or AST results 2 times normal levels should prompt consultation with a pediatric hepatologist. The importance of regular transaminase evaluation is critical as hepatic inflammation can quickly progress to frank fibrosis. In a review of children diagnosed with NALFD in Toronto, Rashid et al [23]. showed a high prevalence (77%) of progression to liver inflammation and fibrosis. This rapid trajectory underscores the importance of follow-up in those children with elevated liver transaminases as end-stage complications can be present in childhood. Particularly concerning is the process starting at a young age even if advanced findings are not yet present. Continued and improved interaction between gastroenterologists and pediatric endocrinologists are needed as the team approach to medical management of patients with T2DM may require more comprehensive medical evaluation including liver biopsies. At our institution, biopsies are typically performed only if the ultrasound suggests cirrhosis. Further longitudinal studies may provide better guidelines regarding the progression of liver disease in relation to the timing of biopsy. Our data also suggest that variations in the management should occur based on ethnicities possibly using different timing of monitoring and goals of treatment. One might more readily refer and/or follow more closely a Hispanic patient with laboratory or imaging studies suggesting NAFLD.

## Conclusions

Our preliminary findings suggest that Hispanic youth with T2DM are more likely to have abnormal liver transaminases, which, in turn, could indicate in a greater risk for developing NAFLD compared to their African-American and non-Hispanic white peers. Although these findings need to be confirmed via prospective studies using more accurate assessments of hepatic health, the increasing rates of NAFLD and T2DM renders our report important to clinicians and researchers working with obese youth.

## Abbreviations

T2DM: Type 2 diabetes mellitus; ALT: Alanine aminotransferase; AST: Aspartate aminotransferase; NAFLD: Non-alcoholic fatty liver disease; BMI: Body Mass Index; ULN: Upper Limit of Normal.

## Competing interests

The authors declare that they have no competing interests.

## Authors’ contributions

OH conceptualized the study, extracted the data, and drafted the manuscript. MN extracted the data and edited the manuscript. GQS conceptualized the study, analyzed the data, and edited the manuscript. All authors read and approved the final manuscript.

## Pre-publication history

The pre-publication history for this paper can be accessed here:

http://www.biomedcentral.com/1471-2431/12/174/prepub
